# Generalized Linear Models for Flexible Parametric Modeling of the Hazard Function

**DOI:** 10.1177/0272989X19873661

**Published:** 2019-09-26

**Authors:** Benjamin Kearns, Matt D. Stevenson, Kostas Triantafyllopoulos, Andrea Manca

**Affiliations:** The University of Sheffield, Sheffield, UK; The University of Sheffield, Sheffield, UK; The University of Sheffield, Sheffield, UK; The University of Sheffield, Sheffield, UK; The University of York, York, UK

**Keywords:** dynamic survival models, fractional polynomials, frailty models, generalized additive models, generalized linear mixed models, splines, survival analysis, time to event

## Abstract

**Background.** Parametric modeling of survival data is important, and reimbursement decisions may depend on the selected distribution. Accurate predictions require sufficiently flexible models to describe adequately the temporal evolution of the hazard function. A rich class of models is available among the framework of generalized linear models (GLMs) and its extensions, but these models are rarely applied to survival data. This article describes the theoretical properties of these more flexible models and compares their performance to standard survival models in a reproducible case study. **Methods.** We describe how survival data may be analyzed with GLMs and their extensions: fractional polynomials, spline models, generalized additive models, generalized linear mixed (frailty) models, and dynamic survival models. For each, we provide a comparison of the strengths and limitations of these approaches. For the case study, we compare within-sample fit, the plausibility of extrapolations, and extrapolation performance based on data splitting. **Results.** Viewing standard survival models as GLMs shows that many impose a restrictive assumption of linearity. For the case study, GLMs provided better within-sample fit and more plausible extrapolations. However, they did not improve extrapolation performance. We also provide guidance to aid in choosing between the different approaches based on GLMs and their extensions. **Conclusions.** The use of GLMs for parametric survival analysis can outperform standard parametric survival models, although the improvements were modest in our case study. This approach is currently seldom used. We provide guidance on both implementing these models and choosing between them. The reproducible case study will help to increase uptake of these models.

In many medical studies, the outcome of interest is the time until an event occurs. Examples include mortality, disease progression, or hospital admission. To aid with decision making, the hazard function is estimated from parametric models. A prominent example is health technology assessment, which aims to quantify both the benefits to patients and the costs a health care system would incur if a treatment were funded.^1^ To allow for fair comparisons across different treatments, it is important that all relevant benefits and costs are quantified, which often requires use of a lifetime horizon.^2^ However, time-to-event (TTE) data with complete follow-up are rarely available. As such, parametric models may be used to extrapolate model outcomes to a lifetime and hence obtain estimates of mean TTE (such as mean survival).^3,4^

Standard 1- and 2-parameter models are available, including the exponential, Weibull, Gompertz, log-logistic, and lognormal models.^5^ However, these models may not be sufficiently flexible to capture complex, time-varying hazards.^6,7^ In the next section, we introduce generalized linear models (GLMs) and show that standard survival models may be expressed as GLMs. This provides insight into the limitations of the standard models: they all impose an assumption of linearity. More flexible parametric models that relax this assumption are required. A number of these have been proposed within the framework of GLMs and their extensions, but to date, they are seldom used to analyze TTE. These are described in the sections titled “Relaxing the Assumption of Linearity” and “Extensions to the GLM,” with an overview in the “Theoretical Comparison of Approaches” section. An application of these is described in the section titled “Empircal Comparison of Approaches,” which demonstrates that the GLM-based models can provide superior within-sample estimates and more plausible extrapolations than standard survival models. Concluding remarks are provided in the “Discussion” section.

This article has 2 aims. The first is to propose the use of GLMs for the analysis of TTE data. This includes flexible GLMs such as fractional polynomials (FPs) and restricted cubic splines (RCS), which are closely related to Royston-Parmar (R-P) models. The second aim is to present generalizations to GLMs: generalized linear mixed models (GLMMs),^8^ generalized additive models (GAMs),^9^ and dynamic generalized linear models (DGLMs).^10,11^

## Analyzing TTE Data within a GLM Framework

### Standard Survival Models as Linear Models

The framework of GLMs extends (generalizes) the standard linear model to response variables with distributions in the exponential family, including normal, Poisson, binomial, gamma, and inverse Gaussian distributions.^12^ An advantage of GLMs is that they provide a unified framework—both theoretical and conceptual—for the analysis of many problems, including linear, logistic, and Poisson regression.^13^ A random variable *Y* belongs to the exponential family of distributions if its probability density (or mass) function can be written as follows:

(1) $$f(y_{t};\theta )=\exp[a(y)b(\theta )+c(\theta )+d(y)],$$

where a(y) and d(y) are functions of the data, whereas b(θ) and c(θ) are functions of the distribution parameter *θ* and assumed to be twice differentiable. Equation 1 may also include other parameters, which are treated as nuisance parameters.^13^ Examples for the normal, Poisson, and binomial distributions are provided in Table 1. For these, a(y)=y.

**Table 1:** Normal, Poisson, and Binomial Distributions as Members of the Exponential Family^a^

Distribution	b(θ)	c(θ)	d(y)
Normal	$\frac{\mu }{\sigma^{2}}$	-$\frac{\mu^{2}}{2\sigma^{2}}-\frac{1}{2}\log(2\pi \sigma^{2})$	$\frac{-y^{2}}{2\sigma^{2}}$
Poisson	logθ	−θ	−logy!
Binomial	$\log(\frac{\pi }{1-\pi })$	nlog(1−π)	$\log\left(\begin{array}{c} n\\ y \end{array}\right)$

a μ and $\sigma^{2}$ are the mean and variance, π is the probability, n is the number of trials, and $\left(\begin{array}{c} n\\ y \end{array}\right)=\frac{n!}{y!(n-y)!}$ is the binomial coefficient.

For a TTE GLM, the observed outcome is the number of deaths during an interval: $y_{t}$. This is linked to the at-risk population at time t (denoted by $\tau_{t}$) using a distribution from the exponential family. Use of the Poisson distribution assumes that $y_{t}=\tau_{t}\times \lambda_{t}$, where $\lambda_{t}$ is the hazard at time t. Alternatively, use of the binomial distribution assumes that $y_{t}=\tau_{1}\times p_{t}$, where $p_{t}$ is the cumulative probability of death. The model specification is as follows^12^:

(2a) $$\mathrm{Observation}\;\mathrm{model}:\;E[y_{t}]=\mu_{t}\times \tau_{t},\;y_{t}\sim \mathrm{exponential}\;\mathrm{family}\;\mathrm{distribution}$$

(2b) $$\mathrm{Response function}:\;\mu_{t}=h(x_{t}^{T}\beta ),$$

where E[·] denotes the expected value, the bold font denotes a vector, and

β is a vector of parameter coefficients to be estimated from the data;

$x_{t}$ is a covariate, assumed known (with transpose $x_{t}^{T}$); and

h() is a one-to-one response function that maps the linear predictor $\left(x_{t}^{T}\beta_{t}\right)$ to $\mu_{t}$. Its inverse is known as the link function and is denoted as g().

Model parameters may be obtained via maximum likelihood estimation. The general expression for the logarithm of the likelihood is

$$\log\;\;L=\sum_{t=1}^{N}L_{t}=\sum_{t=1}^{N}y_{t}b(\theta_{t})+\sum_{t=1}^{N}c(\theta_{t})+\sum_{i=t}^{N}d(y_{t}),$$

where N is the number of time intervals. For the Poisson and binomial models, this becomes

(3a) $$\mathrm{Poisson}:\;\log\;L=\sum_{t=1}^{N}\left[y_{t}\log\;(\theta_{t})-\theta_{t}-\log\;(y_{t}!)\right]$$

(3b) $$\mathrm{Binomial}:\;\log\;L=\sum_{t=1}^{N}\left[y_{t}\log\;\left(\frac{\pi_{t}}{1-\pi_{t}}\right)+n_{t}\log\;(1-\pi_{t})+\log\;\left(\begin{array}{c} n_{t}\\ y_{t} \end{array}\right)\right].$$

In summary, a GLM may be specified by 3 components:

1. The distribution from the exponential family, as defined in equation 1;
2. the response (or link) function; and
3. the covariate vector.

For survival analyses, options for $\mu_{t}$ include the (cumulative) survival function, its complement the (cumulative) failure function, the hazard function, and the cumulative hazard function; see references 5 and 14 for more details. Depending on the specification, we can express standard survival models as a linear model: $\mu_{t}=\beta_{0}+\beta_{1}x_{t}$. Table 2 provides these specifications. The log-logistic and lognormal distributions have a cumulative function as their outcome. It would not be sensible to model such an outcome as a constant value, which demonstrates why there is no single-parameter special case of these models. In contrast, the Weibull and Gompertz distributions model a noncumulative outcome, so it is possible to model this as a single value, resulting in the exponential model.

**Table 2:** Specification of Standard Survival Models as Generalized Linear Models

$\mu_{t}$	Distribution	Response Function	Covariate	Model
Hazard	Poisson	Exponential	None	Exponential
Hazard	Poisson	Exponential	Time	Gompertz
Hazard	Poisson	Exponential	Log(time)	Weibull
Cumulative failure	Binomial	Logistic	Log(time)	Log-logistic
Cumulative failure	Binomial	Inverse probit	Log(time)	Lognormal

An important aspect of survival data is that there is typically censoring of observations. Censoring occurs because for standard models, the outcome is the time of the event occurring, and for some individuals, the event is not observed (so it is censored). Within the GLM formulation, time changes from being the outcome to a covariate, so there are no censored observations. Information on censoring is included by calculating the “at-risk” sample and including this information in the model. For models with a binomial distribution, there is an explicit parameter for the sample size. For models with a Poisson distribution, information on the sample size may be incorporated as an “offset” term.

### Limitations with Linearity

The assumption of linearity may not always be realistic. For example, for overall survival, the hazard of all-cause mortality will increase over time due to patient aging. In contrast, frailty effects may result in in a decrease in disease-specific mortality over time (as those with an increased hazard will die sooner, leaving those with a lower hazard). The impact of treatment on survival may also vary over time: there may be an initial elevated risk of death due to adverse events, treatment-related toxicities may increase other-cause mortality over time, and treatment stopping rules and trial inclusion criteria may have an effect.^15^ These considerations motivate the need for more flexible survival models, which are considered within the GLM framework in the next 2 sections.

## Relaxing the Assumption of Linearity

We briefly describe flexible models that may be applied to survival data within a GLM framework. More details are provided in the Supplementary Appendix. Without loss of generality, y is used to denote either a random variable or the observed data.

### Fractional Polynomials

FPs represent the outcome as a sum of polynomial terms; increasing the number of terms (the order of the FP) increases the flexibility of the model. A closed-test procedure may be used to identify the order. For a single variable, an *i*th-order FP is defined as

(4) $$E(y_{t})=\mathrm{FP}(i)=\beta_{0}+\sum_{j=1}^{i}\beta_{j}x^{p_{j}}$$

where the set of powers $p_{j}$ is prespecified and may include fractional powers (hence, the name FPs). FPs include linear models as special cases, so depending on specification, they may include one of the standard models from Table 2. Some limitations with FPs are that they may not have sufficient power to detect nonlinearity, and they can be sensitive to extreme values in the data. This sensitivity occurs because FPs are global models: β values are assumed to be constant over time.

### RCS and R-P Models

A cubic spline represents a continuous function as a series of piecewise cubic polynomials,^14^ hence relaxing the assumption of global time effects. Model flexibility is based on the number of piecewise intervals (equivalently, the number of “knots”). For extrapolation, the cubic polynomial from the last interval may be used, or it may be restricted to a linear function: this latter assumption results in an RCS. An example specification is provided in the Supplementary Appendix.

R-P models use RCSs but not in the GLM framework. Typically, the outcome is the log cumulative hazard, which is monotonic. However, model estimates are not guaranteed to be monotonic, so implausible values may result.

As they are not global models, splines may overfit local “noise” in the data,^16^ and there is in general no closed-test procedure for choosing between different models.

## Extensions to the GLM

This section provides a brief overview of extensions to GLMs, with more details in the Supplementary Appendix.

### Generalized Linear Mixed Models

A GLMM extends the GLM by incorporating random effect terms, which can help to quantify the impact of unmeasured covariates and provide more realistic estimates of uncertainty. An example of an FP(2) with a random effect (denoted by $b_{t}$) is

$$E(y_{t})=\mathrm{FP}(2)=\beta_{0}+b_{t}+\beta_{1}x^{p_{1}}+\beta_{2}x^{p_{2}},\;b_{t}\sim N(0,\psi^{2}).$$

GLMMs are also referred to as *frailty* models.^17^ In theory, any GLM may be extended by adding a random term as shown above. The main limitation with GLMMs is that as the random effects are not observed, there may be difficulties in model specification and parameter estimation.

### Generalized Additive Models

A GAM is a GLM in which 1 or more of the covariates are modeled as a set of basis functions.^18^ For example, a univariate GAM is defined as

$$E(y_{t})=\sum_{j=i}^{q}b_{j}(t)\beta_{j}=f(t\;),$$

where $b_{j}(t)$ is the *j*th basis function and q is the dimension of the basis function. Higher values of q result in more flexible models. Both FPs and RCSs may be viewed as GAMs. The main extension provided by a GAM is that model complexity is penalized during parameter estimation (via shrinkage of the β). GAMs with a cubic spline basis have theoretical justification as being approximate “smoothest interpolators”^9;^ see the Supplementary Appendix for more details. Limitations of GAMs will depend on the basis function used. For example, if a spline is used, the limitations of these will still apply.

### Dynamic GLMs and Dynamic Survival Models

In a DGLM model, coefficients (β) are allowed to vary over time. When applied to TTE data, DGLMs are known as *dynamic survival models* (DSMs).^19^ Specification is (compare with equation 2)

(5a) $$\mathrm{Observation}\;\mathrm{model}:\;E[y_{t}]=\mu_{t}\times \tau_{t}\;y_{t}\sim \mathrm{exponential}\;\mathrm{family}\;\mathrm{distribution}$$

(5b) $$\mathrm{Response}\;\mathrm{function}:\;\mu_{t}=h(x_{t}^{T}\beta_{t})$$

(5c) $$\mathrm{Transition}\;\mathrm{model}:\;\beta_{t}=F\beta_{t-1}+\zeta_{t}$$

(5d) $$\mathrm{Initialconditions}:\beta_{0}\sim MVN(b_{0},Z_{0}),$$

where MVN denotes a multivariate normal distribution, F is a function describing how the coefficients evolve over time, and $\zeta_{t}$ is an error term (see the Supplementary Appendix for further details). DGLMs may be viewed as combining GLMs with time-series methods. In particular, parameter estimates may be based on minimizing the error of within-sample extrapolations. This makes these models particularly appealing when the primary objective of the analysis is extrapolation. The main limitations with DGLMs are identifying suitable initial values and convergence of algorithms to estimate model coefficients.^19,20^

## Theoretical Comparison of Approaches

Five different modeling approaches were considered: FPs, splines, GAMs, GLMMs, and DGLMs. The frailty terms from a GLMM may be combined with either of the other 4 models. The following prompts are provided to aid with choosing between the different approaches.

**What is the primary objective of the analysis?** If the main objective is in generating extrapolations, this implies the use of a DGLM, as this is the only one of the models for which parameter estimation is based on minimizing forecasting error. If instead the main objective is to provide estimates of the observed data, then any of the approaches may be used.
**FPs or spline-based models?** Spline-based models may be preferred on theoretical grounds, as being approximate smoothest interpolators, whereas there are a number of limitations with the use of FPs (see the Supplementary Appendix). This suggests the use of a spline-based model in preference to an FP within a GLM framework, with the latter as a form of sensitivity analysis.
**To penalize during or after estimation?** Parameter estimation with a GAM automatically penalizes for model complexity, which helps to avoid overfitting. Alternatively, information criteria may be used. There are a number of different information criteria that could be used, whereas GAMs have a specific objective function. The choice between these is likely to be study specific: sometimes there may be good reasons to use a specific information criterion, whereas in other cases, the more automated approach of a GAM may be preferred. For both approaches, it is not possible to use significance tests to choose between model specifications.
**Are there any subject matter considerations?** For example, there may be reason to believe that there are important unmeasured confounders, which suggests incorporating random effects. Or it may be thought that there will be important local fluctuations in this hazard, which suggests the use of either a spline or dynamic model in preference to the global FPs.

## Empirical Comparison of Approaches

### Data Set

We used a freely available data set to demonstrate both the limitations of assuming linearity and the use of more flexible models. Analyses were performed in R; the code used is available as supplemental material. Hence, the case study is fully reproducible.

The data were on the survival of individuals following a diagnosis of breast cancer and from a study conducted by the German Breast Cancer Study Group.^21,22^ Individuals with primary node-positive breast cancer were recruited between July 1984 and December 1989. Events were defined as either cancer recurrence or death (from any cause). Data were available for 686 individuals, of whom 299 experienced an event during follow-up. The maximum follow-up was 7.28 years, with a mean follow-up of 3.08 years. The use of GLMs required that individual-level data were restructured in the form of life tables. Samples of the individual-level data and the corresponding (monthly) life table are provided in Tables 3 and 4, respectively. For Table 3, an event indicator of 1 denotes that an event occurred (otherwise, the indicator is 0, and the outcome is time to censoring).

**Table 3:** Sample of the Breast Cancer Data

Patient ID	Outcome Time (y)	Event Indicator
1	0.0219	0
15	0.1973	1
220	1.9562	1
221	1.9644	0
678	6.7288	1
686	7.2849	0

**Table 4:** Data from Table 3 Restructured for Poisson Regression

Month	Sample Size	Events (μ)	Censorings	At risk (τ)	Hazard (λ)
(0, 1)	686	0	7	682.5	0
(1, 2)	679	0	3	677.5	0
(2, 3)	676	1	4	674	0.001
(22, 23)	477	5	3	475.5	0.011
(23, 24)	469	7	4	467	0.015
(24, 25)	458	8	12	452	0.018
(87, 88)	1	0	1	0.5	0

**Figure 1:**
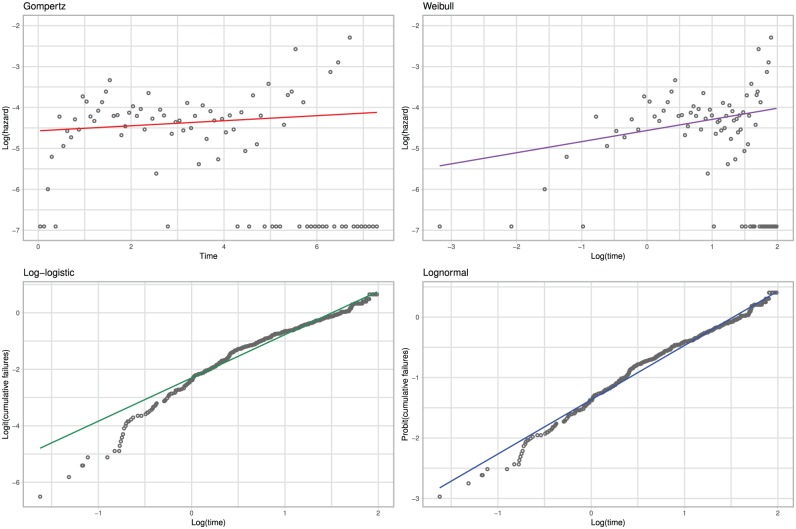
Breast cancer case study: observed and modeled hazard.

As described in the “Limitations with Linearity” section, the assumptions of linearity imposed by standard 2-parameter survival models may be unrealistic. To highlight this, we show model estimates against the observed data in Figure 1 for each model (the 1-parameter exponential model is not shown, as it would be appropriate only if both the Weibull and Gompertz estimates had no slope). The specification of the *x*- and *y*-axes is such that the model estimates form a straight line. Figure 1 shows that the linear estimates generally provide a poor visual description of the data, with the best description arising from the lognormal model.

### Methods

We considered 5 broad classes of models.

#### FP models

We considered FP(2) models, with the complexity of the chosen model based on the closed-test procedure, and the chosen powers based on minimizing Akaike’s information criterion (AIC).

#### Generalized linear mixed models

We fit FP models as described above, but we also included frailty terms.

#### Spline-based models

Both RCS models and GAMs were considered. For the RCS model, between 1 and 5 internal knots were considered, with the choice based on minimizing AIC. For the GAM, we considered 2 approaches to selecting the dimension of the basis function: one used a fixed (arbitrary) value of 11 (v1), and the other was based on minimizing AIC (v2). These 2 approaches were considered, as some penalization for overfitting is included during model fitting, so it is unclear if model choice based on AIC is required. For all models, the knots were placed at equally spaced percentiles of the observed uncensored death times.^21^

#### Dynamic models

We examined 3 specifications: local level, local trend, and local level with global trend. There was no need to base model choice on minimizing AIC (as the data used to estimate the model parameters are separate from the objective function, which is based on minimizing 1-step ahead forecasts).

#### Standard survival models

Eight survival models were considered: exponential, Weibull, Gompertz, gamma, log-logistic, lognormal, generalized gamma, and generalized F. Results are displayed for the 3 best-fitting models (based on AIC). Note that the generalized gamma and generalized F models have 3 and 4 parameters, respectively, and so are more flexible than the standard survival models of Table 2.

The above choice of models was designed to be representative but not exhaustive of the variety of different approaches possible. All of the models used the natural logarithm of time as the only covariate of interest (with the exception of the Gompertz, which used time). All of the GLM-models assumed a Poisson distribution with an exponential response function.

### Goodness of Fit

Goodness of fit (GoF) measures how well the statistical model describes the observed data. It should be distinguished from predictive ability, which measures how well the model predicts external data (such as future observations). One measure of GoF is AIC, which is defined as

(6) −2logL+2k,

where L is the model likelihood and k is the number of parameters in the model.^23^ Because the likelihood is multiplied by a negative number, lower AIC values are to be preferred.

A number of variants on AIC have been proposed.^23,24^ An empirical study by Hyndman and colleagues^24^ compared 5 GoF measures and noted that they all performed similarly. Further, Burnham and Anderson^23^ noted that the AIC has strong theoretical motivation, whereas Jackson and colleagues^25^ noted that the AIC is preferable when models are used to represent complex phenomena (such as survival processes). Because it has both empirical and theoretical support, the AIC shall be used in this article. Any GoF measure should be used in combination with subject-matter considerations. In addition, estimates of the hazard function were visually compared with the observed hazard function.

The AIC measures GoF to the observed data. It is unknown if models with a good within-sample fit provide good extrapolations.^14^ To measure the extrapolation performance of the models, we split the data set into 2 parts. The first part considered events occurring within the first 3 years, censoring all events after 3 years (half of the sample were at risk of an event at 3 years). Extrapolation performance was defined as the sum of squared errors (SSE) between the model estimate of the hazard and the observed hazard (calculated for monthly intervals) for the remaining follow-up:

(7) $${\left({\hat{\lambda }}_{t}-\lambda_{t}\right)}^{2},t\in \{37\;\mathrm{to}\;88\;\mathrm{months}\}.$$

## Results

**Figure 2:**
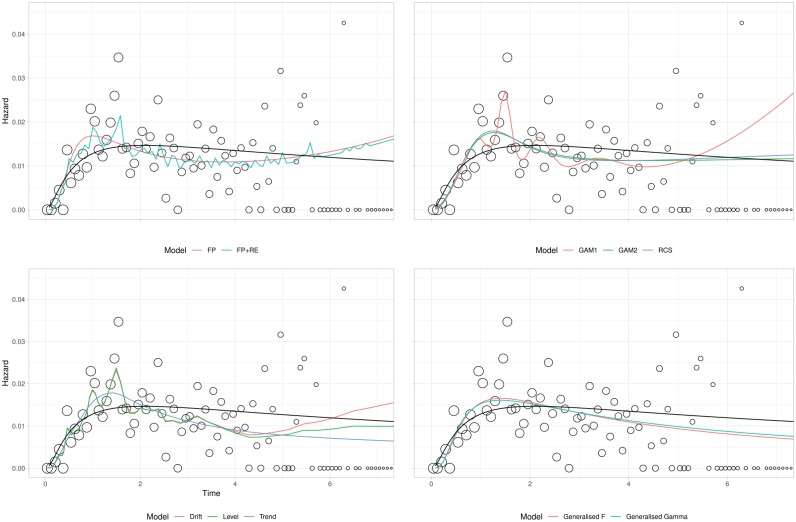
Breast cancer case study: observed and modeled hazard. FP, fractional polynomial; RE, random effects; RCS, restricted cubic spline; GAM, generalized additive model; Gen, generalized. Hollow circles represent observed data; sizes are proportional to the denominator. For all panes, the lognormal is included in black. Three observations are removed: see Figure 3 for these.

**Figure 3:**
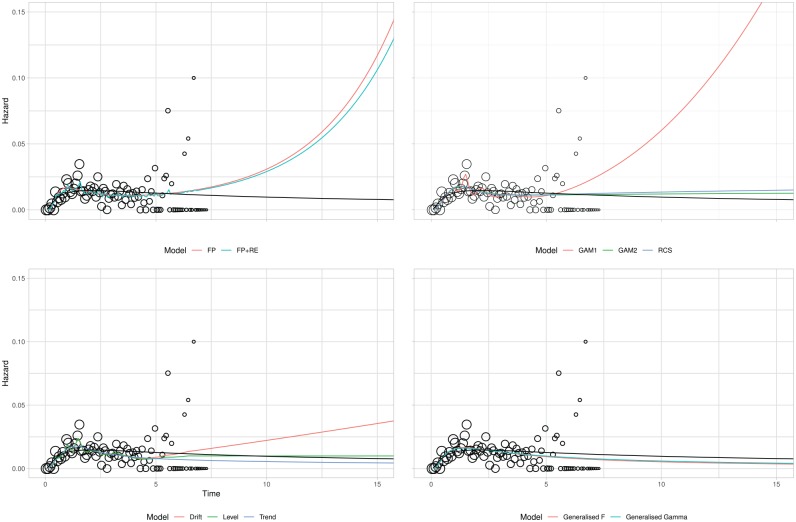
Breast cancer case study: extrapolated hazards. FP, fractional polynomial; RE, random effects; AR, autoregression; RCS, restricted cubic spline; GAM, generalized additive model; Gen, generalized. For all graphs, the lognormal is included in black.

Table 5 provides GoF values for each model and estimates of lifetime mean life expectancy. Two AIC values are provided: one using the entire data set the other using the first 3 years. The number of parameters is provided as a measure of model complexity: the 2 GAMs do not have an integer number of parameters, as parameter effects are shrunk during model estimation. Plots of the estimated hazard function for each model are displayed in Figure 2 for the observed data. Corresponding extrapolations are given in Figure 3. As the best-fitting 2-parameter standard survival model (based on all the available data), the lognormal is provided as a black reference line on all panes.

**Table 5:** Breast Cancer Case Study: Log-Likelihood and Information Criteria for the Models

Model	Log-Likelihood	Parameters	AIC: Full Data	AIC: Years 1–3	SSE^a^: Years 4–7	Life Expectancy
Local level	−142.72	3	291.45	168.48	3.84	37.62
Local level with drift	−142.09	4	292.19	180.25	18.58	23.41
GAM v2	−150.63	3.84	308.94	172.08	4.01	37.12
RCS	−150.55	4	309.10	172.12	4.05	35.46
GAM v1	−144.05	10.66	309.42	173.89	3.81	14.13
Generalized Gamma	−153.03	3	312.06	175.31	3.78	43.40
FP with random effects	−152.13	4	312.27	173.54	4.25	15.70
FP	−153.42	3	312.84	172.51	4.29	15.40
Generalized F	−152.97	4	313.94	174.40	4.01	43.87
Local-level local trend	−152.36	5	314.71	180.68	3.76	41.61
Lognormal	−157.55	2	319.11	179.42	3.73	40.64

AIC, Akaike’s information criterion; FP(2), second-order fractional polynomial; GAM, generalized additive model; RCS, restricted cubic splines; SSE: sum of squared errors (×10 000).

a For derivation of SSE values, see the “Goodness of Fit” section.

### Within-Sample GoF

All of the more flexible models provide lower AIC values than the lognormal, although in general, differences between values are small and cannot be tested for statistical significance. Visually, all of the models provide a good fit to the observed data in Figure 2, although there is variation in the degree to which local fluctuations are captured.

Of the 11 models, the lowest AIC values arose from 2 DSMs. However, the third DSM had the highest AIC of all the flexible models. This suggests that the extension to dynamic models can lead to an improved GoF, but there is no guarantee that this will always occur. The next best AIC values arose from the 3 spline-based models, all of which had very similar GoF. However, the 2 approaches to GAM estimation did result in markedly different models: the one with automated fitting was more complex (with almost 3 times as many parameters) than the one based on minimizing AIC while also providing a better absolute fit (based on log-likelihood).

Of the 3 standard survival models, the 2 generalized models (gamma and F) provided similar GoF, and both improved on the 2-parameter models. Fit for the 2 FPs was similar to that for the generalized gamma and generalized F survival models and lower than that for the spline models. The inclusion of random effects had a negligible impact on the AIC.

Flexible parametric modeling of the hazard provides insight into how it varies over time. The GAM (v1) and DSMs were slightly better at capturing local fluctuations in the hazard rate. This is most notable at approximately 1 and 1.5 y. However, as the most flexible models considered, there is a danger that these local fluctuations represent noise. If this is the case, then the best-fitting models may be overfitting the data, with no guarantee that this will lead to improved extrapolations.

### Extrapolation GoF

When fitting the 11 models to the first 3 years, the ranking of the models was generally the same as for the full data set, with the local-level model providing the lowest AIC and the lognormal one of the highest. An exception is the DSM with drift, which changes from having the second lowest AIC to the second highest. GoF to the observed data did not predict extrapolation performance. For example, both the lognormal and local trend models had the highest AIC values but the lowest SSEs. As with the AIC values, in general there was little difference between SSE values. An exception is the DSM with a drift, which provided poor extrapolations as it predicted an increasing trend.

In general, the results in Table 5 demonstrate that there is little difference between the competing models, both for within-sample and extrapolated GoF. However, Figure 3 shows that resulting extrapolations (beyond the full data follow-up) can vary markedly by model. Differences begin at about 4 years and are likely to be due to the small patient numbers. For example, at 5 y, the sample size at risk is 113; at 6 years, it is 34; and at 7 y, it is 3. When choosing between the models, it is very important to assess the plausibility of the extrapolations with clinical experts, noting the outcome definition used. For this case study, the mean age of the sample is 53 y, and the outcome is either cancer recurrence or death from any cause. The mean survival for German women of this age was 32.6 y in 2000 (the oldest year for which there are data). This acts as an upper bound on the likely survival of this sample, as women with breast cancer are likely to have worse survival than the age-matched general population, and cancer recurrences would further reduce the estimated survival. Of the 11 models considered, only the 4 that predicted an increasing extrapolated hazard (DSM with drift, GAM with default settings, both FPs) gave a lifetime mean survival less than this.

## Discussion

A wide variety of flexible parametric models may be used to analyze and extrapolate TTE data within a GLM framework, along with its extensions to GAMs, GLMMs, and DGLMs. These include FPs, spline-based models, and DSMs. An advantage of the GLM-based models over standard survival models is that they can be made arbitrarily flexible as required to match the complexity of the observed hazard function (for example, increasing the order of an FP or the number of knots in an RCS). In contrast, to obtain more complex standard survival models, different specifications are required (such as moving from a Weibull to a generalized gamma model). Further, 2 of the GLM extensions (GAMs and DGLMs) penalize for overfitting as part of parameter estimation,^9,20^ thus removing much of the subjectivity over model choice. To our knowledge, this is the first time that all of these approaches have been compared at both a theoretical and an applied level, with recommendations to aid in choosing between the models.

The case study demonstrated that it is straightforward to perform a TTE analysis within a GLM framework and that results are at least as good as, and often superior to, those from standard survival models. However, differences in GoF were typically small, and in this example, there was no relationship between within-sample GoF and extrapolation performance. A strength of the case study is that we considered a variety of different statistical models, some of which are currently infrequently used in survival analyses.^3,19^ The fully reproducible nature of the case studies shall help to increase the uptake of these more advanced methods.

There were marked differences in the extrapolations from each model and hence estimates of lifetime mean survival. Using external evidence, only the extrapolations from 1 each of the DSMs and GAMs along with both FPs were plausible, whereas the best 3 standard survival models all provided implausible extrapolations. This highlights a further benefit of the GLM approach, as it increases the potential to identify models that simultaneously provide good within-sample fit and plausible extrapolations. Formally incorporating such evidence is an important area of ongoing research.^26,27^ However, this task is often nontrivial. For example, external data sets may exist, but they may not be fully generalizable to the decision problem. This could be due to differences in the patient population, the health care system, or the time period. Hence, this external data set may need to be adjusted, and assumptions shall be required about how the observed data relate to the external data set.

Parametric analysis of TTE data typically has up to 2 objectives: to obtain a parsimonious description of the observed data and/or to predict outcomes for the unobserved future (extrapolation). More work is required into the relative strengths and weaknesses of the alternative models in both settings. For example, for the best-fitting FP model, inclusion of random effects had a negligible impact on the AIC. Further research is required to see if this is a general phenomenon, or if more nuanced modeling would lead to a more substantive improvement in fit, or if these enhancements would be beneficial for other observed hazard patterns. The case study also highlights that a within-sample measure of GoF cannot be used to choose between models for extrapolation, as has been observed previously.^27–29^ The case study expands on these findings as it compares global models (FPs and survival models), piecewise models (spline-based models), and local models (DSMs). Further work on model choice when used for extrapolation could build on the the work of forecasting competitions.^30^

The case study had limitations. First, we compared models based on AIC (within-sample) and SSE (extrapolations). We were not able to test the differences for statistical significance. For AIC, there is some guidance on what differences may be important, but this holds only for nested models.^23^ While the more flexible models generally improved within-sample fit, they did not improve extrapolation performance. In addition, for many analysts, use of the more flexible models will come at an additional cost, as there will be a need to understand both the theoretical details (strengths and limitations) of the method and how to implement the model. The guidance in the “Theoretical Comparison of Approaches” section and the reproducible case study should help to reduce these costs, although they will still be a factor when choosing between the difference models.

The use of a single case study may also be viewed as a limitation. It is unclear if the (generally) superior GoF provided by DSMs and GAMs generalizes to other settings. The results for the 3 DSMs illustrate an important caution against generalization: if only the 2 DSMs without a local trend were considered, DSMs would provide the best-fitting models. In contrast, if only the DSM with a local trend were considered, we would conclude that their fit is not as good as that of the spline-based models. The GoF of the DSM with drift also varied markedly between using the full data set and using the first 3 years of data. More experience with these different models and their performance for different sample sizes and follow-up times is required before firm conclusions can be made about which (if any) will provide more accurate estimates.

## Conclusion

Parametric modeling of the hazard function allows for predictions of future outcomes. Standard survival models may be insufficiently flexible to reflect the complexities of observed hazard patterns. The GLM framework and its extension to GAMs, GLMMs, and DGLMs can provide insight into the structure of standard 1- and 2-parameter models and their assumptions of linearity. In addition to providing more flexible models (as we have demonstrated here), it also allows for a rich class of model specifications via different combinations of the outcome, distribution, and response function, although this comes at the cost of needing to understand how and when to implement these models. We have provided guidance to aid in the choice between these models. Further, spline-based GLMs provide a useful alternative to R-P models: with appropriate response function, these models cannot estimate implausible negative hazards, unlike R-P models. A motivating and fully reproducible case study has demonstrated that these currently underused approaches can sometimes provide better GoF and more plausible extrapolations than standard survival models.

## Supplemental Material

Appendices_online_supp – Supplemental material for Generalized Linear Models for Flexible Parametric Modeling of the Hazard Function Click here for additional data file. Supplemental material, Appendices_online_supp for Generalized Linear Models for Flexible Parametric Modeling of the Hazard Function by Benjamin Kearns, Matt Stevenson, Kostas Triantafyllopoulos and Andrea Manca in Medical Decision Making

## Supplemental Material

BC_Analysis_online_supp – Supplemental material for Generalized Linear Models for Flexible Parametric Modeling of the Hazard Function Click here for additional data file. Supplemental material, BC_Analysis_online_supp for Generalized Linear Models for Flexible Parametric Modeling of the Hazard Function by Benjamin Kearns, Matt Stevenson, Kostas Triantafyllopoulos and Andrea Manca in Medical Decision Making
