# Supplementary material for: Generalized Linear Models for Flexible Parametric Modeling of the Hazard Function
Source: Med Decis Making. 2019 Sep 26;39(7):867–78. doi: 10.1177/0272989X19873661 (PMC6843612; doi:10.1177/0272989X19873661)
Supplement: Appendices_online_supp – Supplemental material for Generalized Linear Models for Flexible Parametric Modeling of the Hazard Function [file Appendices_online_supp.pdf]

Journal Title
XX(X):1–13

©The Author(s) 0000
Reprints and permission:
sagepub.co.uk/journalsPermissions.nav

DOI: 10.1177/ToBeAssigned
www.sagepub.com/
SAGE

Generalised linear models for flexible parametric modelling of the hazard function: Appendices

Benjamin Kearns[1], Matt Stevenson[1], Kostas Triantafyllopoulos[1], and Andrea Manca[2]

1 Flexible generalised linear models

This section describes flexible models that may be applied within a GLM framework, and hence may be used for the analysis of survival data. To aid interpretation, the focus is on situations where the only covariate of interest is time (a common occurrence in HTA), although extensions to additional covariates are straight-forward. Subsequent sections describe extensions to GLMs.

1.1 Fractional polynomials

FPs have been developed to provide a systematic framework for identifying and modelling non-linear effects of a continuous variable [1]. The degree of flexibility of an FP is defined by its order (how many terms it includes). For a single variable, an ith order FP is defined as:

$$E(y_t) = \text{FP}(i) = \beta_0 + \sum_{j=1}^{i} \beta_j x^{p_j} \tag{1}$$

where typically the powers p_j are chosen from the set {-2, -1, -0.5, 0, 0.5, 1, 2, 3}, with x^0 denoting $\log x$. If the power of a term is duplicated then the duplicated term is multiplied by $\log x$. Hence,

[1]The University of Sheffiled
[2]The University of York

Corresponding author:
Benjamin Kearns, HEDS, ScHARR. University of Sheffield Regent Court, 30 Regent Street Sheffield, S1 4DA.
Email: b.kearns@sheffield.ac.uk

for example, a second order FP of a single variable may be written as $\text{FP}(2) = \beta_0 + \beta_1 x^{p_1} + \beta_2 x^{p_2}$ if $p_1 \neq p_2$ and $\text{FP}(2) = \beta_0 + \beta_1 x^{p_1} + \beta_2 x^{p_2} \log x$ if $p_1 = p_2$. It is possible to consider FPs with order > 2, and values of p_j other than those described. However, in practice these extensions are unlikely to lead to improvements in goodness of fit [2]. Similarly, other possible values for p_j may be considered, but may not. For any given order, the powers to use may be based on minimising the AIC. To choose the order of FP, the following (approximate) closed test procedure may be used [1]:

- Overall association of the outcome with time, comparing FP(2) model with model omitting time (Non-significant result = stop testing, do not include time in model).
- Evidence for non-linearity, comparing FP(2) model with a model that is linear in time (Non-significant result = stop testing, use a linear model: power = 1).
- Simpler or more complex non-linear model, comparing FP(2) model with FP(1) model (Significant result = use FP(2) model, non-significant = use FP(1) model).

Comparisons are performed using likelihood ratio tests, with a pre-specified significance level. A limitation of FPs is that, when using the eight powers described above, the continuous variable has to be > 0. This is not a restriction for TTE data, but it can be a problem if transformations of time are used. For example, the generalised F (and hence its special cases, which include the generalised Gamma, Weibull, lognormal and log-logistic amongst others[3]) use the logarithm of time, which can be negative. Of the eight powers described above, only five may be used with negative values: {-2, -1, 1, 2, 3}. Further, it is not possible to handle repeated powers, which would result in taking the lograithm of the covariate. Hence, for log time there are five FP(1) models and 10 FP(2) models. For time as a covariate (no log transformation) there are eight FP(1) models and 36 FP(2) models. If the closed test procedure rejects a non-linear model, and a Poisson GLM is used, then the chosen model will be the same as a standard TTE model. A linear model in time is analogous to a Gompertz model, a linear model in log-time is analogous to a Weibull model, and a model without time as a covariate (and so just an intercept) is analogous to an exponential.

1.1.1 Limitations Whilst FPs are flexible and relatively parsimonious, they have some limitations:

- Insufficient power to detect non-linearity [1]
- Inability to model a variety of functional forms including logarithmic functions and 'threshold effects' [4]
- Lack of invariance with respect to the coding used for covariates: any transformations such as centering or scaling can lead to different FPs being chosen [5]
- Reduced options for modelling covariates that can be positive.
- Sensitivity to extreme values in the data [6]

The last limitation can be a particular issue if extreme values occur near the end of follow-up time, with subsequent implications for extrapolation.

1.2 Restricted cubic splines and Royston-Parmar models

A cubic spline represents a continuous function as a series of piecewise cubic polynomials [7]. These cubic polynomials are restricted to join (have the same value) at a set of 'knots'. The complexity of the cubic polynomial representation depends on how many knots are used. There will always be at least two knots, known as 'boundary' knots. Any additional knots are known as 'interior' knots and occur between the boundary knots. Hence for k interior knots the knot locations are $\xi_{\min} < \xi_1 < \cdots < \xi_k < \xi_{\max}$, with the boundary knots being $\{\xi_{\min}, \xi_{\max}\}$. Cubic splines also have continuous first and second derivatives.
A RCS is further restricted to be linear beyond the boundary knots (that is, before $\xi_{\min}$ and after $\xi_{\max}$) [8]. A spline has several alternative formulations. One common description for an RCS is as a truncated power series [8, 4]:

$$\begin{aligned} E(y_t) &= \beta_0 + \beta_1 x + \beta_2 V_1(x) + \cdots + \beta_{k+1} V_k(x) \\ \text{with } V_j(x) &= (x - k_j)_+^3 - \psi_j (x - k_{\min})_+^3 - (1 - \psi_j)(x - k_{\max})_+^3, \quad j \in \{1, \ldots, k\} \\ \text{and } \psi_j &= (k_{\max} - k_j)/(k_{\max} - k_{\min}) \\ \text{where } (x - a)_+ &= \max(0, x - a) \end{aligned} \tag{2}$$

where the $V_j(x)$ are referred to as basis functions. Alternative bases are formed by b-splines or p-splines (which are a function of b-splines). B-splines are numerically more stable than the truncated power series basis. However, in practice this advantage is usually negligible, and b-splines cannot be used to generate extrapolations, whereas the truncated power series basis can [4].
In the absence of any interior knots, an RCS is a linear function. Hence, as previously noted, standard survival models may be obtained as special cases of RCSs. For extrapolations beyond $k_{\max}$ the RCS is a linear function of x.

For a Poisson GLM, the outcome is the hazard rate. An alternative application of RCSs, outside the GLM framework, is with an R-P model, for which the log cumulative hazard is the outcome [8]. This is motivated by noting that the hazard function may be more noisy than the cumulative hazard function. This increased noise is most likely near the end of follow-up time, due to small numbers. These 'end effects' may induce spurious artefacts in the spline function. A drawback of directly modelling the cumulative hazard is that this is monotonic (it cannot decrease), but this property cannot be expressed by a simple set of constraints on the parameters of the spline. Hence it is possible that implausible functions may be fitted, as a modelled decreasing cumulative hazard would imply that there is a period for which the hazard rate is negative. An example of implausible fit to real-data is provided in Figure 4b of [9]. Further developments of the R-P model are described by Lambert and Royston [10, 7].

The model specification for a RCS includes both the number of internal knots, and also their placement. It has been suggested that the former decision is more important than the latter and that a maximum of three internal knots is usually sufficient, as larger values may lead to over-fitting the data [8, 4]. When introducing the R-P model, the authors also suggested that knots be placed at equally-spaced intervals of the uncensored event times (with the boundary knots placed at the first and last of the observed uncensored event times)[8]. The choice of how many interior knots to use is typically based on minimising an information criteria.

1.2.1 Limitations The main limitation with RCS is that, due to their flexibility, they may over-fit local 'noise' in the data [11]. The can also be 'data hungry', as they can produce biased results in small samples [12]. In addition, unlike FPs, there is in general no closed test procedure for choosing between different models. One exception is that linear models are always nested within more complex models, so a test for non-linearity is possible.

2 Generalised linear mixed models

The presence of unmeasured variables can lead to heterogeneity (overdispersion) in the outcome that is in excess of what is implied by a model. This will lead to underestimating uncertainty in covariate effects [13]. To remedy this, GLMs may be extended by incorporating random effect terms, resulting in GLMMs. These terms have a mean of zero and a variance parameter, usually unknown. Frailty refers to the feature that individuals have unequal conditional probabilities of experiencing the outcome of interest, given these random (frailty) terms. [14]. Frailty models are appropriate when data are collected on multiple levels; for example individuals may be clustered within families, or they may be clustered by centre within a multicentre clinical trial. Because of this, frailty models may also be referred to as multi-level models [15].

Frailty models may be used to analyse TTE data outside a GLMM framework [13]. However, with the GMLM framework, frailty models may be modelled as mixed models, which have been extensively researched [16, 17]. Mixed models are so-called as they contain both fixed effects and random effects. Fixed effects occur in a standard GLM; for these effects (parameter coefficients) are assumed to apply (are fixed) for all individuals. In contrast, random effects are allowed to vary across individuals. To avoid identifiability issues, it may be assumed that the random effects are drawn from a zero-mean Normal distribution (other zero-mean distributions such as t-distribution may also be used).

As an example, consider a fixed-effects 2nd-order FP which, if powers are not duplicated, may be written as:

$$E(y_t) = \mathrm{FP}(2) = \beta_0 + \beta_1 x^{p_1} + \beta_2 x^{p_2}$$

When analysing TTE data with a GLM, individual observations relate to individual time intervals. As there is only one observation per time-interval, a GLMM with a random intercept may be used. That is, the linear predictor in any time-interval t is increased or decreased by an additive amount b_t. For example:

$$E(y_t) = \mathrm{FP}(2) = \beta_0 + b_t + \beta_1 x^{p_1} + \beta_2 x^{p_2}, \quad b_t \sim N(0, \psi^2) \tag{3}$$

The above extension is not specific to FP models. That is, any fixed-effects GLM may be extended by adding a random intercept as shown above.

2.1 Limitations

As the frailty terms are not observed, it may not be possible to identify them from the data, which can lead to problems with model specification and estimation. [13, 18]. A further limitation with the use of frailty models is that it is unclear how random effect terms should be extrapolated. One option is to fit a model to the estimated random effects and use this to predict future values. Alternatively the last estimated random effect may be carried-forwards.

3 Generalised additive models

A generalised additive model (GAM) may be viewed as a GLM in which a covariate x is replaced by a linear combination of a finite set of smooth functions $b_1, \ldots, b_q$ [19]. For example, a univariate GAM is defined as:

$$E(y_t) = \sum_{j=i}^{q} b_j(t)\beta_j = f(t) \tag{4}$$

Where $b_j(t)$ is the jth basis function, and q is the dimension of the basis function. Modelling the effect of a covariate as a sum of basis functions results in extremely flexible models, with more flexible models arising as more functions are added to the basis. The basis functions may be non-parametric, which results in semi-parametric GAMs [19]. However, the focus here is on parametric basis functions, and hence parametric GAMs [20].

Of the parametric basis functions, use of RCSs is of particular interest. Model estimation for a GAM is different to that for a GLM. For the latter, model complexity may be penalised after model fitting via the use of information criteria, such as the AIC. For a GAM, model complexity is included in the objective function to be minimised. For example, for Normally distributed data this would be [20]:

$$\sum_{t=1}^{n} \Big(y_t - f(t)\Big)^2 + \Lambda \int \Big(f''(t)\Big)^2 dt \tag{5}$$

The integral for the second term is taken over the range of the data and penalises the wiggliness of function, to avoid over-fitting the data. The first term in equation (5) quantifies model goodness of fit. In this example it is the sum of squared errors, but a more general representation uses a negative log-likelihood, as is used in a GLM. This leads to the following objective function [21]:

$$-2\sum_{t=1}^{n} \mathcal{L}_t\Big(y_t; f(t)\Big) + \Lambda \int_{-\infty}^{\infty} \Big(f''(t)\Big)^2 dt \tag{6}$$

As with AIC, the objective function measures the trade-off between model goodness of fit and model complexity. The key difference is that for GAMs the complexity of the model is estimated during the model-fitting process, and is not a post-hoc choice. For a GAM, Λ quantifies this trade-off. As $\Lambda \to \infty$ the estimated function tends towards a straight line. For $\Lambda = 0$ there is no penalty on the regression spline (so the resulting model will be equivalent to a RCS model if a RCS basis is used).

There are two components to model estimation, as the smoothing parameter Λ and the model coefficients β are estimated separately [20]. One approach to estimating the degree of smoothing is based on a generalisation of leave-one-out cross-validation, known as generalised cross-validation. For given values of Λ, model coefficients are estimated based on penalised likelihood estimation [22].

As with standard GLM models, GAMs may also be extended to incorporate random effects. The resulting models are known as generalised additive mixed models [23]. This extension does not result in any concepts additional to those outlined in section 2.

3.1 Choice of basis function

A full RCS (with a knot at each unique value of t) has the desirable property of being a 'smoothest interpolator' [20]. That is, for all functions that are continuous over the range of the data and also have an absolutely continuous first derivative, a full RCS will minimise equation (6) [21]. In addition, a RCS is usually flexible enough to adequately describe most functions. An alternative choice of basis function is to use polynomials. Here the jth basis function is defined as [20]:

$$b_j(t) = t^{j-1} \tag{7}$$

In comparison with equation (2), the basis for polynomials is much simpler. The first two terms are also the same as for a RCS, and so the use of polynomial basis functions also includes standard models such as the Weibull and Gompertz as special cases. However, the use of polynomial basis functions is not recommended [20, 4]. Unlike RCSs, there is no guarantee that polynomials will provide an adequate fit across the entire range of the data. Further, the fit in a local region of the data can be strongly affected by the characteristics of the data in other regions. Also, polynomials are unable to provide adequate descriptions of a number of functions, such as those including 'threshold effects' or logarithmic functions. It should be noted that FPs (section 1.1) do not follow the hierarchical rule that is used when constructing polynomial basis functions. Indeed, FPs include fractional powers, which are not in the definition of a polynomial basis function in equation (7). Hence, unlike R-P models, FPs cannot be viewed as a special case of a GAM. As such the limitations that apply to the use of polynomial basis functions do not necessarily apply to the use of FPs.

3.2 Regression splines

A full smoothing spline has as many parameters as there are data points. These full splines may be approximated by regression splines, which are no longer 'smoothest interpolators', but are computationally easier to fit [20]. A simulation study comparing full splines with their regression approximation found that the approximation led to a superior fit (to the known 'true' function) [24]. The author attributed this counter-intuitive finding to the fact that regression splines are also less likely to overfit the data (or equivalently, the full splines was modelling random variation).

When using regression splines, the basis dimension has to be specified (it is not part of model estimation). In practice the smoothing parameter Λ in equation (6) has a larger impact on model complexity than the choice of basis dimension. Hence the basis dimension acts as an upper-bound for model complexity. Provided it is sufficiently large, model results are typically insensitive to the choice of basis dimension [20]. A slight exception is that larger basis dimensions lead to a larger set of candidate models, which can sometimes affect model choice.

3.3 Limitations

When using RCSs as basis functions, GAMs share the same limitation in that they require sufficiently large datasets, and they may over-fit local noise in the data [24]. However, the use of a penalized objective function slightly mitigates this latter limitation.

4 Dynamic generalised linear models and dynamic survival models

A DGLM extends a GLM by allowing the model coefficients to vary as a smooth function of time. These may be used to analyse TTE data, resulting models are known as dynamic survival models (DSMs). As an illustrative example, consider the exponential model: $\log(\mu_t) = \beta_0$. This may be interpreted as a global level model as the outcome is set to a fixed level for all times. This assumption can be relaxed by allowing β_0 to vary over time. Hence $\log(\mu_t) = \beta_{0,t}$, giving a local-level model.

Without further restrictions, this may lead to an over-fitting model. For example, setting $\beta_{0,t} = y_t$ would give a perfect fit to the observed data. However, this will over-fit the data, so to avoid this the following restriction is used:

- When estimating the local level at time t, only evidence available prior to time t is used. This can include previous estimates of the local level, previous covariate values, and previous observations.

More formally, at time t, let the prior available evidence be denoted by the history of prior outcomes: $\mathcal{H}(y)_t = \{y_1, y_2, \ldots, y_{t-1}\}$, the history of prior coefficient estimates $\mathcal{H}(\beta)_t = \{\beta_1, \beta_2, \ldots, \beta_{t-1}\}$, and the history of prior covariates $\mathcal{H}(x)_t = \{x_1, x_2, \ldots, x_{t-1}\}$. Then the estimate of the local level at time t is obtained as a function of all these histories $\mathcal{H}(y, \beta, x)_t, = \mathcal{H}_t$ for simplicity.

This estimate is a forecast (extrapolation) from the evidence at time $t-1$ to time t. As such, it is referred to as a *one-step-ahead forecast*.

The approach of DSMs represents a small, but significant change in estimation from previously described approaches. For these, a model estimate of y_t (denoted by $\hat{y}_t$) used all the observed data. Such an estimate is known as a *smoothed estimate*, and models minimised the error of smoothed estimates. In contrast, DSMs minimise the error of (within-sample) forecasts. Hence the choice between a DSM and a GLM (or GAM) is likely to be based on if the analyst wants to minimise the error of smoothed estimates or the error of forecasts.

Some further comments on the use of the forecasts are required.

- It shall be assumed that the Markov property is valid [25]; so $\hat{y}_{t|1:t-1} = \hat{y}_{t|t-1}$. In addition, sometimes an estimate at time t uses y_t. This is no longer a one-step ahead forecast, but is referred to as a *filtered estimate*, denoted as $\hat{y}_{t|1:t} = \hat{y}_{t|t}$.

- When estimating the one-step ahead forecast $\hat{y}_{t+1|t}$, evidence is available for all the prior one-step ahead forecasts (which are a function of the β_t). However, evidence is also available on the previous outcomes. This may be used to estimate the accuracy of the previous forecasts, and this knowledge used to improve estimates of $\hat{y}_{t+1|t}$. Hence the one-step ahead forecast $\hat{y}_{t+1|t}$ is based on prior filtered values, and so the histories $\mathcal{H}(\beta)_t$ include filtered values, not one-step ahead forecasts.

- Specification of a DSM requires initial (starting) estimates. There will be no histories to inform these, so they may be based on external data, expert opinion, or via a heuristic [26].

A limitation with DSMs is that model estimation can be difficult: either due to computational issues, or if the use of approximate methods is inappropriate [27, 28]. Extrapolations from a DSM are determined by the chosen model specification, which is described in the following subsections.

4.1 Model specification

The general model specification for a DGLM (or DSM) is [21]:

$$\text{Observation model: } E[y_t] = \mu_t \quad y_t \sim \text{exponential family distribution} \tag{8a}$$

$$\text{Response function: } \mu_t = h(x_t^T \beta_t) \tag{8b}$$

$$\text{Transition model: } \beta_t = F\beta_{t-1} + \zeta_t \tag{8c}$$

$$\text{Initial conditions: } \beta_0 \sim MVN(b_0, Z_0) \tag{8d}$$

where MVN denotes a multivariate Normal distribution, F is a transition matrix for the coefficients over time, and the error term (ζ_t), also referred to as an *innovation*, is assumed to be an independent and identically distributed series with $\zeta_t \sim N(0, Z_t)$, where Z_t is a variance-covariance matrix of the model coefficients. As before, for a Poisson GLM, the outcome is the number of number of events in an interval (which, combined with knowledge of the at-risk population, can be used to derive the hazard rate). Of note, the covariate time does not appear within the model specification. That is, no assumptions are made about the relationship between time and the hazard rate. Instead it is assumed that there exist latent states (the βs), such as an average value (level) and trend - either of which may vary over time.
Three types of DLM are of particular interest. Two are the local level and local trend models, which may be interpreted as zero-order and first-order Taylor series approximations, respectively [26]. The third is the local level global trend model, also referred to as a local level with drift model. These are defined below.

4.1.1 Local level models

$$\begin{aligned} E(y_t) &= \beta_t \\ \beta_t &= \beta_{t-1} + \zeta_t \end{aligned}$$

this type of model is referred to as a random-walk model. Extrapolations of the y_t are a constant value, equal to the last-estimated local level. That is, the extrapolated value h time steps into the future, given observed data up to time T is $\hat{y}_{T+h|T} = \beta_T$,

4.1.2 Local trend (linear) models

These may be defined as:

$$\begin{aligned} E(y_t) &= x^T \beta_t = [1, 0] \begin{bmatrix} \beta_{1t} \\ \beta_{2t} \end{bmatrix} = \beta_{1t} \\ \beta_t &= \begin{bmatrix} \beta_{1t} \\ \beta_{2t} \end{bmatrix} = \begin{bmatrix} 1 & 1 \\ 0 & 1 \end{bmatrix} \begin{bmatrix} \beta_{1,t-1} \\ \beta_{2,t-1} \end{bmatrix} + \begin{bmatrix} \zeta_{1t} \\ \zeta_{2t} \end{bmatrix} \\ \beta_{1t} &= \beta_{1,t-1} + \beta_{2,t-1} + \zeta_{1,t} \\ \beta_{2t} &= \beta_{2,t-1} + \zeta_{2,t} \end{aligned}$$

where $\beta_t = [\beta_{1t}, \beta_{2t}]^T, \zeta_t = [\zeta_{1t}, \zeta_{2t}]^T$. The formula for β_{1t} may be written down recursively as:

$$\begin{aligned}
\beta_{1t} &= \beta_{1,t-1} + \beta_{2,t-1} + \zeta_{1,t} \\
&= \beta_{1,t-2} + 2\beta_{2,t-2} + \zeta_{1,t} + \zeta_{1,t-1} + \zeta_{2,t-1} \\
&= \beta_{1,t-3} + 3\beta_{2,t-3} + \zeta_{1,t} + \zeta_{1,t-1} + \zeta_{1,t-2} + \zeta_{2,t-1} + 2\zeta_{2,t-2} \\
&= \ldots \\
&= \beta_{1,0} + t\beta_{2,0} + \sum_{i=1}^{t} \zeta_{1i} + \sum_{j=1}^{t-1} j\zeta_{2,t-j}
\end{aligned}$$

where the first two terms are a linear function, and the last 2 terms are random error. As such, β_{1t} represents the local level, which is a linear function of t. The parameter β_{2t} may be interpreted as the growth rate (local trend). Extrapolations are a linear function of the last-estimated local level and local trend values: $\hat{y}_{T+h|T} = \beta_{1T} + h\beta_{2T}$.

4.1.3 Local level global trend (drift) models This may be viewed as a special case of the local trend model, with $\zeta_{2,t} = 0$. A single trend β_2 is then estimated based on all the data. This estimate of the trend will be more stable than the one estimated locally as towards the end of follow-up the number of observations is smallest. However, this increased stability is at the loss of flexibility, as the estimate of trend is fixed and so does not vary with time.

4.2 Likelihood specification

The likelihood for a DSM may be written as the product of likelihoods for individual time-periods j (denoted by $\mathcal{L}_j$), with t_j denoting the jth time-period. Following Hemming and Shaw [29] the following two variables are introduced:

$$\delta_{ij} = \begin{cases} \delta_i & \text{if } t_i^* \leq t_j \\ 0 & \text{if } t_i^* > t_j \end{cases} \qquad t_{ij} = \begin{cases} t_i & \text{if } t_i^* \leq t_j \\ t_j & \text{if } t_i^* > t_j \end{cases} \tag{9}$$

hence for an individual who experienced an event, $\delta_{ij} = 1$ for all time periods up-to and including the time period which included the event. In all other situations it is zero. The indicator t_{ij} is the observed survival time up-to and including the time period which included the observation. Subsequently it is set equal to the time interval. The likelihood for a Poisson DSM is then:

$$\mathcal{L} = \prod_{j=i}^{N} \mathcal{L}_j = \prod_{j=i}^{N} \prod_{i=i}^{\tau_j} \exp(-[t_{ij} - t_{j-1}] e^{x_t^T \beta_j}) e^{x_t^T \beta_j \delta_{ij}} \tag{10}$$

where N is the number of time-intervals, and τ_j is the set of individuals who have a survival time $\geq t_j$. When equal interval widths are used, this is the same likelihood as for a Poisson GLM with an offset (see Section 5).

4.3 Limitations

The flexibility of allowing model coefficients to vary as a function of time can lead to problems with both convergence, and specification of initial estimates [30, 31, 29, 28].

5 Likelihood estimation

5.1 Parametric survival models

Let d_i be an event indicator which $= 1$ if t_i is an observed survival time and $= 0$ if t_i is a right-censored observation (hence the true survival time will be greater than t_i). The likelihood is then [32]:

$$\mathcal{L}_i = \left\{ \prod_{i:\ d_i=1} f_i(t_i) \prod_{i:\ d_i=0} S_i(t_i) \right\} \tag{11}$$

For individuals with an observed event their probability density function contributes to the likelihood. Individuals with censored times contribute their cumulative survivor function (as their probability density function is not fully observed but it is known that they survived up to time t_i). The log-likelihood contribution for the ith patient is:

$$\begin{aligned} \log \mathcal{L}_i &= \log \left\{ f(t_i)^{d_i} S(t_i)^{1-d_i} \right\} \\ &= d_i \log\{f(t_i)\} + (1 - d_i) \log\{S(t_i)\} \end{aligned} \tag{12}$$

The following identities may be used to derive an expression of the likelihood purely in terms of the hazard function [33]: $S(t) = 1 - \int_0^t f(u)du$ and $f(t) = h(t) \times S(t)$. This gives:

$$\begin{aligned} \log \mathcal{L}_i &= \log \left\{ h(t_i)^{d_i} S(t_i) \right\} \\ &= d_i \log\{h(t_i)\} - \int_0^{t_i} h(u)du \end{aligned} \tag{13}$$

5.2 Equivalence between a Poisson GLM with an offset and a Poisson DSM

5.2.1 Poisson GLM with an offset. The probability density function is:

$$f(y_i; \tau_i\theta) = \frac{\tau_i \theta^{y_i} e^{-\tau_i\theta}}{y_i!}$$

The likelihood $\mathcal{L}$ is equal to the sum of the y_i. Taking logarithms and re-arranging gives:

$$\begin{aligned} \mathcal{L} &= \prod \left(\frac{\tau_i \theta^{y_i} e^{-\tau_i\theta}}{y_i!} \right) \\ \log \mathcal{L} &= \sum \left[\log \left(\frac{\tau_i \theta^{y_i} e^{-\tau_i\theta}}{y_i!} \right) \right] \\ &= \sum \left[\log(\tau_i \theta^{y_i}) + \log(e^{-\tau_i\theta}) - \log(y_i!) \right] \\ &= \sum \left[y_i \log(\tau_i\theta) - \tau_i\theta - \log(y_i!) \right] \\ &= \sum \left[y_i \log(\tau_i) + y_i \log(\theta) - \tau_i\theta - \log(y_i!) \right] \end{aligned}$$

The first and last terms do not include the parameter θ. Thus, for a given dataset, the first and last terms will always be the same, and so may be eliminated from the likelihood when

comparing different models. This gives the likelihood:

$$\log \mathcal{L} = \sum [y_i \log(\theta) - \tau_i \theta] \tag{14}$$

5.2.2 Poisson DSM. Consider the Poisson DSM likelihood for any given time-period.

$$\mathcal{L}_j = \prod_{i=i}^{\tau_j} \exp(-[t_{ij} - t_{j-1}]e^{z\beta_j})e^{z\beta_j\delta_{ij}}$$

Assume that intervals are all of the same width, so that the term $[t_{ij} - t_{j-1}] = 1$ and may be omitted. Taking logarithms:

$$\begin{aligned}
\log \mathcal{L}_j &= \sum_{i=i}^{\tau_j} \log\left[\exp(-e^{z\beta_j})e^{z\beta_j\delta_{ij}}\right] \\
&= \sum_{i=i}^{\tau_j} \log[\exp(-e^{z\beta_j})] + \log(e^{z\beta_j\delta_{ij}}) \\
&= \sum_{i=i}^{\tau_j} -e^{z\beta_j} + z\beta_j\delta_{ij} \\
&= d_j z\beta_j - \tau_j e^{z\beta_j}
\end{aligned}$$

where d_j is the observed number of events in the jth time-interval, hence $= y_j$. Let $\theta_j = e^{z\beta_j}$. Then:

$$\log \mathcal{L}_j = y_j \log(\theta_j) - \tau_j \theta_j \tag{15}$$

which is the same as applying equation (14) to a single time-period.

References

[1] Royston P and Sauerbrei W. *Multivariable model-building: a pragmatic approach to regression anaylsis based on fractional polynomials for modelling continuous variables*, volume 777. John Wiley and Sons, 2008. ISBN 0470770783.

[2] Binder H, Sauerbrei W and Royston P. Comparison between splines and fractional polynomials for multivariable model building with continuous covariates: a simulation study with continuous response. *Statistics in Medicine* 2013; 32(13): 2262–2277.

[3] Peng Y, Dear KB and Denham J. A generalized f mixture model for cure rate estimation. *Statistics in medicine* 1998; 17(8): 813–830.

[4] Harrell Jr FE. *Regression modeling strategies: with applications to linear models, logistic and ordinal regression, and survival analysis.* Springer, 2015. ISBN 3319194259.

[5] Peixoto JL. A property of well-formulated polynomial regression models. *The American Statistician* 1990; 44(1): 26–30.

[6] Magee L. Nonlocal behavior in polynomial regressions. *The American Statistician* 1998; 52(1): 20–22.

[7] Royston P and Lambert PC. *Flexible parametric survival analysis using Stata: beyond the Cox model*. United States of America: Stata Press, 2011.

[8] Royston P and Parmar MK. Flexible parametric proportional-hazards and proportional-odds models for censored survival data, with application to prognostic modelling and estimation of treatment effects. *Statistics in medicine* 2002; 21(15): 2175–2197.

[9] Gibson E, Koblbauer I, Begum N et al. Modelling the survival outcomes of immuno-oncology drugs in economic evaluations: A systematic approach to data analysis and extrapolation. *PharmacoEconomics* 2017; : 1–14.

[10] Lambert PC and Royston P. Further development of flexible parametric models for survival analysis. *Stata Journal* 2009; 9(2): 265.

[11] Sauerbrei W, Royston P and Binder H. Selection of important variables and determination of functional form for continuous predictors in multivariable model building. *Statistics in medicine* 2007; 26(30): 5512–5528.

[12] Remontet L, Uhry Z, Bossard N et al. Flexible and structured survival model for a simultaneous estimation of non-linear and non-proportional effects and complex interactions between continuous variables: Performance of this multidimensional penalized spline approach in net survival trend analysis. *Statistical methods in medical research* 2018; : 0962280218779408.

[13] Govindarajulu US, Lin H, Lunetta KL et al. Frailty models: applications to biomedical and genetic studies. *Statistics in medicine* 2011; 30(22): 2754–2764.

[14] Collett D. *Modelling survival data in medical research (Third Edition)*. CRC press, 2015. ISBN 1498731694.

[15] Clark T, Bradburn M, Love S et al. Survival analysis part iv: further concepts and methods in survival analysis. *The British Journal of Cancer* 2003; 89(5): 781.

[16] Molenberghs G and Verbeke G. Models for discrete longitudinal data, 2005.

[17] Fitzmaurice G, Davidian M, Verbeke G et al. *Longitudinal data analysis*. CRC Press, 2008. ISBN 142001157X.

[18] Rodriguez G. Statistical issues in the analysis of reproductive histories using hazard models. *Ann N Y Acad Sci* 1994; 709: 266–79.

[19] Hastie T and Tibshirani R. *Generalized additive models*. Wiley Online Library, 1990. ISBN 0471667196.

[20] Wood SN. *Generalized additive models: an introduction with R*. CRC press, 2017. ISBN 1498728375.

[21] Fahrmeir L and Tutz G. *Multivariate statistical modelling based on generalized linear models*. Springer Science and Business Media, 2013. ISBN 1475734549.

[22] Wood SN. Stable and efficient multiple smoothing parameter estimation for generalized additive models. *Journal of the American Statistical Association* 2004; 99(467): 673–686.

[23] Baayen RH, van Rij J, de Cat C et al. Autocorrelated errors in experimental data in the language sciences: Some solutions offered by generalized additive mixed models. *arXiv preprint arXiv:160102043* 2016; .

[24] Wood SN. Thin plate regression splines. *Journal of the Royal Statistical Society: Series B (Statistical Methodology)* 2003; 65(1): 95–114.

[25] Kedem B and Fokianos K. *Regression models for time series analysis*, volume 488. John Wiley and Sons, 2005. ISBN 0471461687.

[26] Hyndman R, Koehler AB, Ord JK et al. *Forecasting with exponential smoothing: the state space approach*. Springer Science and Business Media, 2008. ISBN 3540719180.

[27] Fahrmeir L and Knorr-Held L. Dynamic discrete-time duration models: Estimation via markov chain monte carlo. *Sociological Methodology* 1997; 27(1): 417–452.

[28] He J, McGee DL and Niu X. Application of the bayesian dynamic survival model in medicine. *Stat Med* 2010; 29(3): 347–60. DOI:10.1002/sim.3795. URL https://www.ncbi.nlm.nih.gov/pubmed/20014356.

[29] Hemming K and Shaw J. A class of parametric dynamic survival models. *Lifetime Data Analysis* 2005; 11(1): 81–98.

[30] Gamerman D. Dynamic bayesian models for survival data. *Applied Statistics* 1991; : 63–79.

[31] Fahrmeir L. Dynamic modelling and penalized likelihood estimation for discrete time survival data. *Biometrika* 1994; 81(2): 317–330.

[32] Jackson CH. flexsurv: A platform for parametric survival modeling in r. *Journal of Statistical Software* 2016; 70(8): 1–33. DOI:10.18637/jss.v070.i08. URL <Go to ISI>://WOS:000384912000001.

[33] Crowther MJ and Lambert PC. A general framework for parametric survival analysis. *Statistics in Medicine* 2014; 33(30): 5280–5297. DOI:10.1002/sim.6300. URL <Go to ISI>://WOS:000346055000006.
